# Internet-Based Support and Coaching With Complementary Clinic Visits for Young People With Attention-Deficit/Hyperactivity Disorder and Autism: Controlled Feasibility Study

**DOI:** 10.2196/19658

**Published:** 2020-12-31

**Authors:** Helena Sehlin, Britt Hedman Ahlström, Ingrid Bertilsson, Gerhard Andersson, Elisabet Wentz

**Affiliations:** 1 Gillberg Neuropsychiatry Centre Institute of Neuroscience and Physiology University of Gothenburg Gothenburg Sweden; 2 Department of Health Sciences University West Trollhättan Sweden; 3 Habilitation & Health, Region Västra Götaland Skövde Sweden; 4 Department of Behavioral Sciences and Learning Linköping University Linköping Sweden; 5 Department of Psychiatry and Neurochemistry Institute of Neuroscience and Physiology University of Gothenburg Gothenburg Sweden

**Keywords:** attention-deficit/hyperactivity disorder, autism, coaching, internet-based intervention, social support

## Abstract

**Background:**

Individuals with attention-deficit/hyperactivity disorder (ADHD) and autism spectrum disorder (ASD) can experience obstacles in traditional health care situations due to difficulties associated with their impairment.

**Objective:**

This controlled study aims to investigate the feasibility of an internet-based support and coaching intervention (IBSC), including 2 weekly chat sessions and 2 complementary clinic visits with coaches over the course of 8 weeks, for adolescents and young adults with ADHD and/or ASD in 2 naturalistic routine care settings.

**Methods:**

Individuals with ADHD and/or ASD aged 15-32 years were recruited in 2 clinical settings, where they received either IBSC (n=24) or treatment as usual (TAU; n=20). Outcome measures included self-report questionnaires assessing quality of life (Manchester Short Assessment for Quality of Life), sense of coherence (Sense Of Coherence 29), self-esteem (Rosenberg Self-Esteem Scale), and anxiety and depressive symptoms (Hospital Anxiety and Depression Scale [HADS] and Montgomery-Åsberg Depression Rating Scale-Self-reported, respectively).

**Results:**

Significant between-group effects were observed in measures of anxiety (HADS) at postintervention (*P*=.02) as well as at the 6-month follow-up (*P*=.004). Significant between-group effects were also noted for depressive symptoms (HADS) postintervention (*P*=.04). The between-group effects were partially explained by a deterioration in the TAU group. A significant increase in self-esteem (*P*=.04) as well as a decrease in anxiety (*P*=.003) at the 6-month follow-up was observed in the intervention group following IBSC. Findings from a qualitative study of the intervention are consistent with the results.

**Conclusions:**

The findings from this study suggest that IBSC holds promise as a feasible complement or alternative to traditional face-to-face health care meetings.

## Introduction

### Background

In the last two decades, neurodevelopmental disorders (NDDs), such as attention-deficit/hyperactivity disorder (ADHD) and autism spectrum disorder (ASD), have increasingly been recognized and diagnosed [1-4]. ADHD is estimated to affect 5% of the child and adolescent population and approximately 2.5% of the adult population [5,6]. The prevalence rates for ASD are estimated to be from 1% to 2.6% [1,7,8]. ADHD and ASD are complex and disabling conditions, which often cooccur with other NDDs [2,9]. If left unmanaged, they can lead to negative consequences, including deterioration of school and work performance, relationships, living situation, self-esteem, and overall quality of life [10-12].

Increased assessment and diagnosis rates for individuals with NDDs have several long-term benefits, including increased access to early treatment for children, which could reduce the effects of core deficits and lessen the occurrence of secondary psychiatric conditions [13]. However, it also calls for efficient treatment and support for adolescents and adults with these conditions. The transition from adolescence into adulthood has been acknowledged as particularly challenging for these individuals [14,15]. This period in life often characterized by a decrease in structure and support at home and in school and increased demands to manage daily living independently. Transitioning from child to adult health care services can also result in a decrease in support for individuals with NDDs [16,17].

Current recommendations for adolescents and adults with ADHD comprise multimodal approaches, including pharmacological interventions, psychoeducation, environmental modifications, and behavioral interventions (eg, cognitive behavioral therapy [CBT]) [10,18,19]. Psychostimulants are often the first-line treatment and have been proven efficacious for core symptoms [10,18], but in approximately 50%, medication alone fails to manage the condition and/or comes with impeding side effects [18,20]. Evidence for CBT remains promising, yet preliminary [18]. Coaching is increasingly recommended as a component of multimodal treatment for adolescents and adults with ADHD [21,22]. Coaching includes individual goal setting, psychoeducation, and a collaborative approach to handle the individual’s main problems. ADHD coaching also relies heavily upon the coach who should possess significant experience and a thorough understanding of the disorder [21,23,24].

Different supports have been developed and tested for ASD, but there is still a great paucity of research within this area. Guidelines promote interventions focusing on communication, interaction, and management of concomitant mental health problems through, for example, CBT [23,24]. In general, guidelines for ASD stress the necessity of making adjustments to the environment, not least to facilitate equal access to health care services [23,24]. There is a lack of studies pertaining to coaching for teens or young adults with ASD. However, a recent review concluded that there is a need for personalized support focusing on daily life problems (eg, mentoring) as opposed to narrow skills training [25].

Internet-delivered treatments have been proven effective for a number of psychiatric disorders [26]. The utilization of chat-based communication in offering psychological support has also increased over the years. A review revealed that chat-based interventions had mostly better or equivalent outcomes when compared with wait-list control and treatment as usual (TAU) [27] and the actual acceptability of this support has been shown to be high, even among people with severe mental health problems [28]. Furthermore, text- and internet-based communication is a familiar part of young people’s everyday life [29].

Individuals with NDDs often struggle with traditional means of support, for example, clinic visits. Nonverbal communication such as eye gaze and gestures can be a challenge for people with ASD, and internet-based support eliminates these issues. Internet-based interventions are also flexible and accessible, which is facilitating for individuals with ADHD who often have trouble with time management and organization. Overall, communication through the internet could prove to be beneficial for individuals with NDDs [30-32]. However, very few interventions exist targeting this specific population. Some data have been published regarding internet-based CBT for ADHD [33] and internet-delivered psychoeducation for adolescents and adults with ASD [34].

### Objectives

Individuals with NDDs comprise a largely heterogeneous group that can experience obstacles in traditional health care. Hence, it is of paramount importance to develop a more needs-based approach to support, especially when transitioning into adulthood. The main objective of this study is to investigate whether an internet-based support and coaching model (IBSC) can be feasible for these individuals. The intervention was first attempted in a small validation study [35] and showed an improved sense of coherence, self-esteem, and quality of life. This study aims to replicate the results and assess the feasibility of the model in 2 naturalistic clinical contexts using a larger sample and a comparison group. Our main hypotheses, based on the previous results, are that an increase in self-esteem, sense of coherence, and subjective quality of life would be observed immediately after the intervention. We also expected symptom reduction for anxiety and depressive symptoms and increased overall quality of life compared with the comparison group.

## Methods

### Study Design

This study (ClinicalTrials.gov Identiﬁer NCT02300597) was designed as a nonrandomized controlled feasibility study. Participants were recruited to either the intervention (IBSC) or the TAU. They were not randomized due to previous experiences regarding recruitment, that is, individuals with ASD have difficulties tolerating uncertainty and were reluctant to participate without knowing the study conditions they were to be allocated. Outcome data in the form of self-report scales were completed before the start of the intervention, after the intervention (8 weeks after baseline for the control group), and 6 months later. Both the intervention and comparison groups received TAU between the 8-week and 6-month follow-up.

### Participants and Recruitment

Participants were consecutively recruited between autumn of 2010 and autumn of 2014 at 2 study centers in the southwest of Sweden: a habilitation center providing assistance for children, adolescents, and adults with NDDs including ASD and an outpatient psychiatric clinic specialized in adults with ASD and ADHD. Participants needed to fulfill the following inclusion criteria: (1) being 15 to 32 years of age, (2) having prior confirmed diagnosis of ADHD, ASD, or both according to the Diagnostic and Statistical Manual of Mental Disorders, 4th Edition (DSM-IV), (3) having access to a computer with internet connection, and (4) no other ongoing support or psychological treatment during the study period (only pertaining to the intervention group). Exclusion criteria were ongoing psychosis, serious and ongoing alcohol and/or substance misuse disorder, major depressive disorder (if an obstacle to conform with the intervention or in better need of other treatment), conduct disorder/antisocial personality disorder, severe dyslexia, and known intellectual disability.

Participants were asked to take part during regular visits at the study sites and received a description of the study, including internet security. Eligibility was confirmed through an interview by a clinical psychologist or psychiatrist using the alcohol/substance use and psychotic disorder modules of the Structured Clinical Interview for DSM-IV Axis I Disorders (SCID I) [36] and the antisocial personality disorder module of the Structured Clinical Interview for DSM-IV Axis II Disorder (SCID II) [37]. Participants completed all outcome measures (see the Instruments section). The Montgomery Åsberg Depression Scale (MADRS) [38] was used both as an outcome measure and to screen for symptoms of depression at baseline.

A total of 31 individuals agreed to participate in the intervention. They were assessed to be eligible for participation and included in the intervention arm. Before the intervention, an individual chose to refrain, leading to a total of 97% (30/31) of individuals in the intervention group.

**Figure 1:**
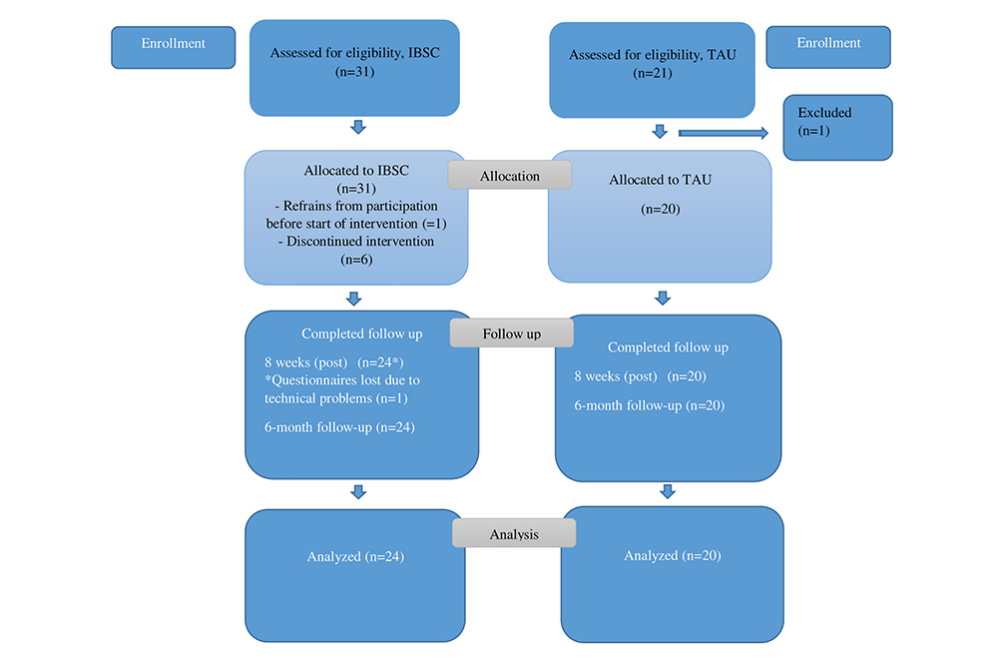
CONSORT (Consolidated Standards of Reporting Trials) flowchart of participants. Regarding TAU, after assessment for eligibility, one individual was excluded due to major depression, leaving 20 to be allocated to TAU. IBSC: internet-based support and coaching; TAU: treatment as usual.

During the course of the study, comparison cases were recruited at each study site. They were matched by age, gender, and NDD diagnoses. In the latter part of the study, some exceptions in terms of matching were made due to recruitment problems. Comparison cases underwent the same procedure with regard to eligibility with respect to inclusion and exclusion criteria. A total of 21 comparison cases were recruited, and all but one (20/21, 95%), who was deemed too depressed, were eligible and included. Figure 1 shows the flow of participants in a CONSORT (Consolidated Standards of Reporting Trials) flowchart. Table 1 provides demographic and clinical characteristics of participants at baseline; only 80% (24/30) completed IBSC; therefore, only 24 were presented.

**Table 1:** Demographics and sample characteristics at baseline.

Demographic		Intervention (n=24^a^)	Treatment as usual (n=20)	*P* value
**Age at inclusion (years)**				
	Mean (SD)	21.0 (5.1)	22.1 (5.1)	.48
	Median (range)	20 (15.0-32.0)	22.0 (15.0-32.0)	.48
**Gender, n (%)**				
	Male	13 (54)	10 (50)	N/A^b^
	Female	11 (46)	10 (50)	>.99
**Diagnosis, n (%)**				
	ASD^c^	9 (38)	7 (35)	N/A
	ADHD^d^	3 (13)	5 (25)	N/A
	ASD+ADHD	12 (50)	8 (40)	.55
**GAF^e^** **score (10-point interval)**				
	31-40	9 (38)	3 (15)	N/A
	41-50	9 (38)	4 (20)	N/A
	51-60	5 (21)	8 (40)	N/A
	61-70	1 (4)	5 (25)	.006
**Study center (1 and 2), n (%)**				
	1	11 (46)	11 (55)	N/A
	2	13 (54)	9 (45)	.76
**Geographical area, n (%)**				
	Urban	13 (54)	17 (85)	N/A
	Rural	11 (46)	3 (15)	.06
**Civil state, n (%)**				
	Married/living with partner	2 (8)	3 (15)	N/A
	In a relationship (not living together)	2 (8)	6 (30)	N/A
	Single	20 (83)	11 (55)	.11
**Living situation, n (%)**				
	One-person household	8 (33)	6 (30)	N/A
	Living with partner and/or children	2 (8)	5 (25)	N/A
	Living with parents and/or siblings	14 (58)	8 (40)	N/A
	Living with friends and/or acquaintances	0 (0)	1 (5)	.28
**Level of education (completed or ongoing)**				
	Has not completed compulsory school^f^	2 (8)	0 (0)	N/A
	Compulsory school	7 (29)	8 (42)	N/A
	Upper secondary school	11 (46)	9 (47)	N/A
	Vocational education (after compulsory school)	0 (0)	1 (5)	N/A
	University	4 (17)	1 (5)	.65
	Missing	0 (0)	1 (5)	N/A
**Occupation, n (%)**				
	Employed	0 (0)	7 (35)	N/A
	Unemployed	6 (25)	3 (15)	N/A
	Student^g^	13 (54)	7 (35)	N/A
	Work experience placement	2 (8)	1 (5)	N/A
	Sick leave	3 (13)	2 (10)	.04
**Support from social services^h^, n (%)**				
	Yes	7 (29)	7 (37)	N/A
	No	17 (71)	12 (63)	.83
	Missing	0 (0)	1 (5)	N/A
**ADHD medication at baseline^i^, n (%)**				
	No	14 (58)	11 (55)	N/A
	Yes	10 (42)	9 (45)	>.99

^a^Only 24 out of 30 in the intervention group completed an internet-based support and coaching intervention; therefore, only 24 are presented in the table.

^b^N/A: not applicable.

^c^ASD: autism spectrum disorder.

^d^ADHD: attention-deficit/hyperactivity disorder.

^e^GAF: Global Assessment of Functioning.

^f^No formal education or terminated compulsory school without complete grades.

^g^All levels of education (eg, compulsory school, secondary school, vocational education, and university studies).

^h^Support can include assistance through the Swedish Act concerning Support and Service for Persons with Certain Functional Impairments (LSS) or from Social Services, for example, so called *contact person*, relief service, or living support.

^i^In all cases but two this was psychostimulant medication.

Overall, the recruitment period spanned over a relatively long period because it took place in routine care settings where the staff had restricted time for the study in relation to parallel practice of routine psychiatric care and/or habilitating interventions. In addition, the participating units were relatively small and each recruitment was immediately followed by coaches engaging in the subsequent intervention (as well as the accompanying administration).

### Intervention and TAU

#### Intervention

**Figure 2:**
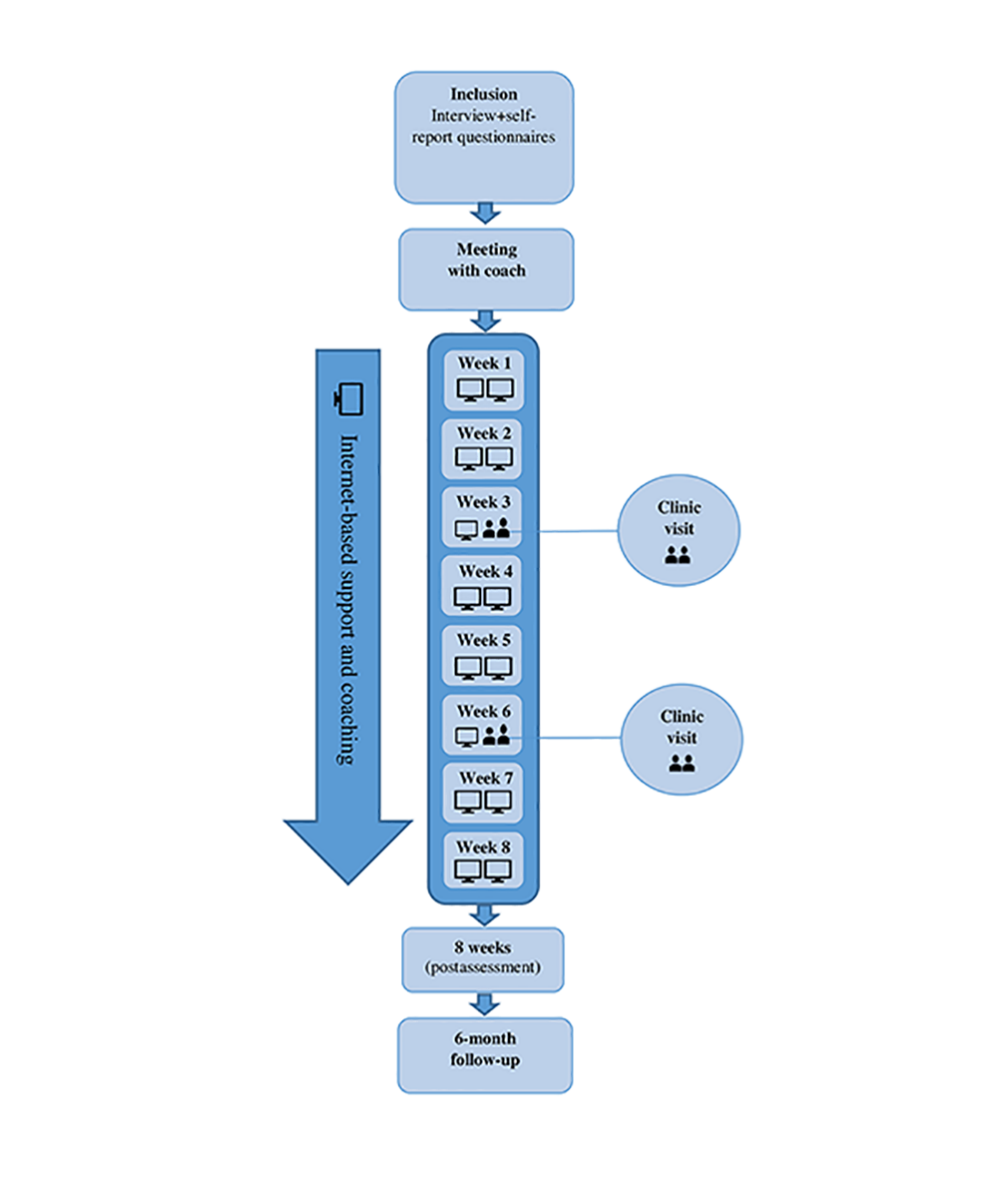
Flowchart of the internet-based support and coaching model. Assessment at 8 weeks (postassessment): assessment was carried out immediately after the end of the 8-week intervention.

The model for IBSC was developed and validated in a study by Wentz et al [35]. The model consists of 8 weeks of internet-based support and coaching mediated through a chat program. It includes 2 chat sessions in a week and 1 individual face-to-face meeting in the clinical setting (replacing chat sessions) during weeks 3 and 6 of the intervention. Each chat session lasted between 30 min and 1 hour. Short communication is also made possible through an email function (Figure 2 provides the IBSC flowchart). The intervention seeks to provide individualized psychoeducation about ADHD and/or ASD as well as support regarding aspects of daily life. The intervention started with a meeting with an appointed coach, with the purpose of becoming acquainted, to inform about the chat program and to help set it up. The coach and participant discussed preliminary areas of focus for the chat sessions as requested by the participant (ie, social interaction, daily routines, stress, study technique, time management). Support and coaching are highly individualized, based on the participants’ expressed needs and requests at each chat session but also continuously readdressing the areas initially agreed on. Coaches validate and acknowledge participants in their experienced difficulties, discuss possible explanations (ie, psychoeducation), empower and encourage participants to find coping strategies, and offer specific advice on managing conveyed everyday life problems. Support and coaching are firmly based on the knowledge and clinical experience of NDDs and is intentionally more focused on everyday support as opposed to highly structured and narrow skill-specific interventions (eg, manualized treatments such as CBT). Coaches were all educated health care professionals, including occupational therapists, clinical psychologists, social workers, and special education teachers, employed at the 2 study sites. All coaches had extensive experience in ASD and ADHD. Before the start of the chat project, coaches were thoroughly informed about the aims and basis of the model, that is, support and coaching in daily life problems as opposed to more serious mental health matters that could require other kinds of help. Every second week, supervision was offered to coaches and research coordinators by the head of the project (the last author; EW). Supervision served as an opportunity to discuss issues concerning the support, model fidelity, and recruitment matters.

#### TAU

TAU comprised any typically occurring treatment, that is, pharmacological treatment and/or psychological treatment, psychosocial support, occupational therapy (interventions pertaining to structure in daily living), and group psychoeducation (with most individuals receiving mainly pharmacological treatment including physical check-ups). Some individuals did not receive any active treatment during the study period (due to difficulties in complying with offered support).

### Instruments

This study used the same instruments as those used in the validation study [35]. All outcome measures were prespecified according to the CONSORT protocol.

#### Primary Outcome Measures

Quality of life (QoL), as assessed by the Manchester Short Assessment for Quality of Life (MANSA), was used as a primary outcome measure in accordance with the validation study. The scale includes 4 objective QoL questions to be answered with *yes* or *no* and 12 subjective questions concerning job, economy, friendships, leisure activities, accommodation, personal safety, living situation, sex life, relationship with family, and physical and mental health [39]. As the study included participants as young as 15 years of age, the scale was modified to exclude the question about sex life. The remaining 11 items were assessed on a 7-point Likert scale (1=negative extreme and 7=positive extreme) and summarized into a total score. The Swedish version of MANSA has been proven to have good reliability and validity in patients with mental illness and has also shown satisfactory reliability in terms of internal consistency [40]. In line with the findings of our validation study, the effects on the first item of the MANSA assessing subjective QoL were analyzed [35].

#### Secondary Outcome Measures

Symptoms of depression and anxiety were measured using the Hospital Anxiety and Depression Scale (HADS) [41]. The HADS consists of 14 questions, with 7 questions constituting 2 subscales: anxiety and depression. Each item is rated on a 4-point scale. The Montgomery-Åsberg Depression Rating Scale-Self-reported (MADRS-S) was also used as a measure of depressive symptoms. It consists of 9 questions that can be scored from 0 to 6 points [38]. In both the HADS subscales as well as in the MADRS-S, a higher score represents a higher symptom burden.

The Sense of Coherence (SOC 29) scale is a life orientation questionnaire built on the concept of salutogenesis and has 3 main components: comprehensibility, manageability, and meaningfulness [42]. The SOC has 29 items, each rated on a 7-point scale where a higher total score represents a better ability to cope with stress and to stay healthy [43]. The Rosenberg Self-Esteem Scale (RSES) was used to measure self-esteem. The questionnaire comprises 10 questions each rated on a 4-point scale. A higher total score reflects better self-esteem [44,45]. Sociodemographic information was collected through a self-report questionnaire (Table 1). Background information on diagnosis, medication, and other received treatment and support was collected through medical records.

#### Global Assessment of Functioning

To assess the participants’ general level of functioning at baseline, all individuals were evaluated retrospectively using the Global Assessment of Functioning (GAF) Scale from DSM-IV- Text Revision [46]. The scale ranged from 0 (severe impairment) to 100 (very high functioning) with descriptions for every 10-point interval considering psychiatric symptoms and social and occupational functioning. The assessment was based on current diagnoses, available sociodemographic information, level of anxiety and depression, and participant responses on the item level for the MANSA. Although not reported in this study, a questionnaire measuring perceived caregiver burden and the individuals functioning in several areas had been completed by next of kin for each participant before the start of the intervention [47]. Information from this questionnaire was similarly used at the item level to assess global functioning. All information except for gender and study arm was summarized into separate case presentations and assessed for GAF score by the main author (HS). Cases were individually and blindly assessed by the last author (EW). As the assessment was conducted retrospectively, a 10-point interval was used, which was deemed more accurate than an exact rating. Interrater reliability was assessed using Cohen kappa (k) and found to be very good (k=0.89). The GAF results are shown in Table 1.

### Statistical Analysis

All statistical analyses were performed using SAS Software version 9.4 (SAS Institute Inc). Differences in baseline characteristics between the intervention and the TAU group (Table 1), as well as between the intervention group and loss to follow-up (an attrition bias analysis is available in Multimedia Appendix 1), were examined using the Fisher exact test for dichotomous variables. The Mantel-Haenszel chi-square test was used for ordered categorical variables, chi-square test was used for nonordered categorical variables, and Fisher nonparametric permutation test was used for continuous variables. For categorical variables, number (%) is presented. For continuous variables, mean (SD)/median (min-max)/numbers of individuals is presented.

For comparisons between groups and to assess the effect of the intervention on primary and secondary outcome measures, the Fisher nonparametric permutation test was used for continuous variables. Analysis of covariance (ANCOVA) models were calculated to adjust for differences in baseline GAF scores, yielding parameter estimates of between-group differences with adjusted means with 95% CI and adjusted *P* values. The same statistical procedure was used to adjust for GAF scores when analyzing the first item of MANSA (overall subjective QoL), which was based on the variable mean score. Primary and secondary outcome measures were predefined before the start of the study and were therefore not adjusted for multiple testing. For comparison within groups on primary and secondary outcome measures, the Wilcoxon signed rank test was used.

Missing data amounted to at most n=1 for all measures at 8 weeks and n=1 for a single scale (HADS) at 6 months. Mean substitution was used to account for missing values at posttreatment and follow-up assessments.

### Ethics Approval

The Regional Ethical Review Board at the University of Gothenburg, Sweden, approved the study (Dnr: 013-08; T364-10; T645-11). Written informed consent was obtained from all participants during the intake interviews. All participants were deemed to have a level of maturity required to provide consent for themselves.

## Results

### Baseline Characteristics

Baseline characteristics presented in Table 1 show that the intervention group and the TAU group were equal in most baseline characteristics. However, the intervention group had a lower mean GAF score than the comparison group. This was also reflected in employment status. Table 2 shows that the intervention and TAU groups were also equal for all outcome measures at baseline (Multimedia Appendix 2).

**Table 2:** Adjusted primary and secondary variables at baseline, after 8 weeks, and 6 months by treatment group.

Variable		Intervention (IBSC^a^; n=24)		Treatment as usual (n=20)		*P* value between groups: adjusted^b^	Mean difference between groups (95% CI); effect size
		Adjusted mean^b^ (95% CI)	*P* value within group	Adjusted mean^b^ (95% CI)	*P* value within group		
**MANSA^c^**							
	MANSA total score baseline	53.7 (49.8 to 57.6)	N/A^d^	52.8 (48.5 to 57.1)	N/A	.77	N/A
	Change baseline to 8 weeks	0.294^e^ (−2.86 to 3.45)	.58	−1.99 (−5.39 to 1.41)	.06	.35	2.28 (−2.57 to 7.13); 0.397
	Change baseline to 6 months	0.247 (−3.19 to 3.68)	.81	−1.84 (−5.63 to 1.96)	.21	.44	2.08 (−3.27 to 7.44); 0.281
**Subjective QoL^f^** **(MANSA)**							
	Subjective QoL baseline	4.55 (4.07 to 5.04)	N/A	4.58 (4.05 to 5.12)	N/A	.94	N/A
	Change QoL baseline to 8 weeks	0.163^e^ (−0.358 to 0.684)	.53	−0.137 (−0.700 to 0.425)	.48	.45	−0.300 (−0.501 to 1.10); 0.279
	Change subjective QoL baseline to 6 months	0.268 (−0.301 to 0.821)	.50	−0.062 (−0.682 to 0.558)	.89	.46	0.321 (−0.554 to 1.20); 0.160
**ROSENBERG^g^**							
	Rosenberg total score baseline	17.3 (15.2 to 19.4)	N/A	17.1 (14.8 to 19.4)	N/A	.90	N/A
	Change total score baseline to 8 weeks	1.57^e^ (−0.06 to 3.20)	.06	0.043 (−1.72 to 1.81)	.79	.23	1.53 (−0.98; 4.04); 0.332
	Change total score baseline to 6 months	1.29 (−0.36 to 2.93)	.04	0.356 (−1.46 to 2.17)	.86	.47	0.930 (−1.64 to 3.49); 0.388
**HADS Anx^h^**							
	HADS Anx baseline	8.02 (6.34 to 9.69)	N/A	7.93 (6.08 to 9.78)	N/A	.95	N/A
	Change HADS Anx baseline to 8 weeks	−0.432^e^ (−1.68 to 0.813)	.44	1.80 (0.45 to 3.14)	.01	.02	−2.23 (−4.15 to −0.31); 0.796
	Change HADS Anx baseline to 6 months	−1.52^e^ (−2.85 to –0.20)	.003	1.55 (0.12 to 2.99)	.02	.004	−3.08 (−5.14 to −1.02); 1.24
**HADS Depr^i^**							
	HADS Depr baseline	3.93 (2.57 to 5.29)	N/A	4.48 (2.98 to 5.99)	N/A	.60	N/A
	Change HADS Depr baseline to 8 weeks	−0.502^e^ (−1.77 to 0.760)	.41	1.58 (0.22 to 2.94)	.06	.04	−2.08 (−4.02; −0.14); 0.675
	Change HADS Depr baseline to 6 months	−0.08^e^ (−1.38 to −1.22)	.80	1.14 (−0.26 to 2.55)	.09	.23	−1.22 (−3.24; 0.79); 0.491
**MADRS-S^j^**							
	MADRS-S total score baseline	12.0 (9.2 to 14.8)	N/A	12.2 (9.1 to 15.3)	N/A	.94	N/A
	Change total score baseline to 8 weeks	−0.103^e^ (−2.66 to 2.45)	.96	1.76 (−1.00 to 4.51)	.23	.34	−1.86 (−5.79 to 2.07); 0.272
	Change total score baseline to 6 months	−0.243 (−3.18 to 2.70)	.62	1.04 (−2.20 to 4.29)	.33	.57	−1.28 (−5.86 to 3.30); 0.374
**SOC^k^**							
	SOC total baseline	125 (116.7 to 133.2)	N/A	121 (112.1 to 130.4)	N/A	.56	N/A
	Change SOC total baseline to 8 weeks	2.02^e^ (−4.32 to 8.35)	.58	−2.77 (−9.60 to 4.07)	.14	.33	4.78 (−4.96 to 14.5); 0.226
	Change SOC total baseline to 6 months	5.81 (−0.62 to 12.2)	.10	−3.39 (−10.5 to −3.71)	.19	.07	9.19 (−0.83 to 19.2); 0.712

^a^IBSC: internet-based support and coaching intervention.

^b^Adjusting for Global Assessment of Functioning interval using analysis of covariance.

^c^MANSA: Manchester Short Assessment of Quality of Life.

^d^N/A: not applicable.

^e^Based on 23 individuals.

^f^QoL: quality of life.

^g^ROSENBERG: Rosenberg Self-Esteem Scale.

^h^HADS Anx: Hospital Anxiety and Depression Scale.

^i^HADS Depr: Hospital Anxiety and Depression Scale.

^j^MADRS-S: Montgomery-Åsberg Depression Rating Scale-Self-reported.

^k^SOC: sense of coherence.

### Dropouts

There were a total of 6 dropouts in the intervention group who had completed at least 1 chat session but did not finalize the full 8 weeks of participation, leaving 80% (24/30) of individuals who completed the IBSC. The reasons stated by these participants for dropping out were related to stress at school or at work and/or difficulties in prioritizing and remembering planned sessions. For one individual, reasons for dropping out are not known. No demographic or outcome variables were significantly associated with the probability of dropout (Multimedia Appendix 1 shows the results of the attrition bias analysis).

### Effect of Intervention

Table 2 illustrates the adjusted primary and secondary outcome measures at baseline, 8 weeks, and 6 months for the 2 groups (Multimedia Appendix 2 provides further details including unadjusted values and standard deviations).

#### Primary Outcome

No statistically significant between-group differences were observed in quality of life (MANSA) from pre- to postintervention (8 weeks; adjusted *P*=.35; adjusted mean difference 2.28; 95% CI −2.57 to 7.13) or at the 6-month follow-up (adjusted *P*=.44; adjusted mean difference 2.08; 95% CI −3.27 to 7.44), and neither were there any significant within-group changes in this measure.

#### Secondary Outcomes

Analyses with ANCOVA adjusted for differing baseline GAF scores revealed a statistically significant between-group difference from baseline to 8 weeks (postintervention) on the HADS (adjusted *P*=.02; adjusted mean difference −2.23; 95% CI −4.15 to −0.31) measuring anxiety symptoms. This difference was explained by a small nonsignificant within-group decrease in anxiety in the intervention group (adjusted mean change score −0.432; 95% CI −1.68 to 0.813) and a significant increase in anxiety in the comparison group (*P*=.01; adjusted mean change score 1.80; 95% CI 0.45 to 3.14). The between-group effect size was large (Cohen *d*=0.80). When comparing the results for anxiety at baseline with those obtained at the 6-month follow-up, there was similarly a statistically significant between-group effect (adjusted *P*=.004; adjusted mean difference −3.08; 95% CI −5.14 to −1.02) with a large between-group effect size (Cohen *d*=1.24). This was explained by a significant decrease in the intervention group (*P*=.003; adjusted mean –1.52; 95% CI −2.85 to −0.20). There was a corresponding significant increase in the comparison group (*P*=.02; adjusted mean 1.55; 95% CI 0.12 to 2.99). For depressive symptoms (according to HADS), there was a significant between-group effect at 8 weeks postintervention (adjusted *P*=.04; adjusted mean difference −2.08; 95% CI −4.02 to −0.14) explained by a nonsignificant decline in depressive symptoms in the intervention group (adjusted mean −0.502; 95% CI −1.77 to 0.760) and a nonsignificant increase in the comparison group (adjusted mean 1.58; 95% CI 0.22 to 2.94) producing a medium-sized between-group effect size (Cohen *d*=0.68).

Finally, there was a significant increase in self-esteem (RSES) for the intervention group at 6 months (*P*=.04; mean change score 1.54, SD 3.59). MADRS-S, measuring depressive symptoms, and MANSA item 1 (subjective QoL) did not improve significantly over time.

## Discussion

### Principal Findings

This study aimed to investigate whether IBSC, including 2 chat sessions in a week and 2 complementary clinic visits over the course of 8 weeks, could be a feasible support option for young people with ADHD and/or ASD. We found that self-esteem increased and symptoms of anxiety decreased in the intervention group at follow-up. Anxiety and depression had improved postintervention compared with TAU, but these findings were partially due to a deterioration in the TAU group. The primary outcome variable, QoL, did not improve over time compared with the TAU group.

The analysis of intervention effects showed that the intervention group experienced significantly increased self-esteem at follow-up. Overall, it seems that living with ASD and ADHD can lead to a number of adverse consequences that, especially when left unmanaged, increase the risk of developing low self-esteem [48-51]. It also appears that self-esteem in individuals with ASD may be less susceptible to, or take longer to change, following treatment interventions with this objective [52,53]. In individuals with ADHD, some results have suggested that psychoeducation might actually worsen self-esteem initially [54,55], whereas a few later studies have been able to observe preserved or even increased self-esteem following such interventions [56,57]. The above results might be seen in the light of individuals with NDDs having trouble producing appropriate coping strategies [52] and the necessity of including elements of strategy building in approaches with the intention of increasing self-awareness [56,57]. The combination of increased self-knowledge and the acquisition of strategies to better handle the struggles of daily living was the main objective of IBSC. In view of the previously outlined research, it seems reasonable that an increase in self-esteem might occur from a longer perspective rather than directly after the intervention. This is supported by results from the validation study where effects on self-esteem were first seen at the 6-month follow-up [35] as well as from the qualitative study of this intervention, in which participants stated improved self-confidence as an experienced long-term effect [31].

Another observed effect in the intervention group was decreased symptoms of anxiety at follow-up. Individuals with NDDs are at great risk of developing anxiety disorders and have exceedingly elevated rates of lifetime psychiatric comorbidity, which adds to their challenges and may overshadow the NDD [58-60]. In the qualitative study of IBSC, participants reported at least short-term calming effects on emotions, in part related to having someone to turn to with thoughts and questions [31]. There was a tendency for reduced anxiety directly following the intervention, and it is possible that the increase in self-esteem observed between the intervention and at the 6-month follow-up for the intervention group was paralleled by a decrease in anxiety at this measuring point. In this study, the comparison group experienced a significant increase in anxiety both at the 8-week and 6-month measuring points. It is important to address the common issue of comorbid anxiety in this population, and it seems that IBSC might have had a positive effect on this measure over time.

We did not, as hypothesized, observe an effect on our primary outcome measure, quality of life (MANSA), nor did we see an effect on sense of coherence (which relates to subjective QoL) [12,61]. QoL was chosen as the primary outcome measure in keeping with the validation study. No significant improvement in the total score of this instrument was seen in the validation study, but there was a significant increase in subjective QoL as well as an increase in sense of coherence at the 6-month follow-up [35]. There are very few studies of SOC or MANSA in relation to ASD or ADHD, making it difficult to draw any safe conclusions about the results. Another study examined ASD traits and how they relate to daily functioning and specific domains of QoL (as measured by MANSA) [62]. The results of this study showed that even though total ASD symptom severity may correlate (negatively) with overall QoL, there is a complexity in that specific ASD traits and symptoms (eg, degree of insistence on sameness) may have different impacts on separate QoL domains. This suggests that it might be difficult to predict if an intervention will have an impact on total QoL scores, as it may also be funneled by secondary variables. In this study, we did not have access to measures of severity regarding specific ASD or ADHD traits; therefore, we cannot rule out that the current sample may have differed from that of the validation study in a way that might have affected the outcome of these measures. In future studies, it might be wise to consider an alternative primary outcome measure. There are several options that could be considered, one being trying a different QoL self-rating scale, for example, the Short Form Health Survey 36 with specific subscales for mental health [63]. Another option could be to use the Clinical Global Impression Scale, which measures the degree of symptom severity and changes over time, as assessed by a blind assessor at baseline, postintervention, and after 6 months [64].

With regard to SOC, there is only one other intervention study, apart from the validation study, which has made use of this instrument with individuals with NDDs [53]. In this study of a randomized controlled trial comparing group CBT with group recreational activity for adults with ASD, there were no observed effects on the SOC directly after the intervention, even with an increased quality of life. When comparing this study to the validation study, total scores on SOC and MANSA at baseline were more or less equivalent. The difference between our sample and that of the validation study is, however, the age range, where the current sample is older, including individuals up to 32 years. It has been proposed that SOC stabilizes and thus fluctuates less after early adulthood [65]. This might be one explanation for the lack of, or unpredictable, effects on this measure compared with the validation study. Moreover, several of the questions in the SOC partially tap into areas that are associated with core difficulties for individuals with ASD, such as the ability to understand social contexts, perceived predictability, and central coherence. This may also have contributed to a certain degree of resilience in this measure.

In a qualitative study of this intervention [31], participants mentioned several features that were experienced as positive about receiving IBSC: an appreciation of the text-based format, always having someone to turn to when experiencing obstacles, and that coaches had sufficient training and experience in the field of NDDs. Participants also described the intervention as easy-going, practical, and supportive in nature [31]. As mentioned in the introduction, it has been suggested that there is a need for support targeting various basic needs for individuals with NDDs [25]. Such support is recommended in the policy programs of several countries although there is a recognized lack of research in this area [25]. In a multicenter, randomized clinical trial by Philipsen et al [66], individual clinical management (CM) was shown to be as effective as cognitive behavioral group psychotherapy on primary outcome measures (ADHD symptoms). CM consisted of nonspecific individual counseling sessions held by a physician with competence within the field of NDD, addressing themes and issues as requested by the patient [66]. This is not unlike the individualized coaching format of the IBSC. In a qualitative study by Giarelli et al [67], perceived bridges and barriers to successful transitioning for adolescents and young adults with ASD were examined. Participants expressed that a supportive mentor was important and helpful for them in a transitioning process. Overall, it seems that individualized support focusing on aspects of daily living offered by someone with sufficient knowledge might be equally important to narrower skills-based approaches for individuals with NDDs. Offering this kind of support through an internet-based contact ameliorates problems related to sensory difficulties, deciphering nonverbal cues and offering flexibility that might be essential in making support accessible for this group of patients.

All these results suggest that the intervention could be a feasible option for young individuals with NDDs. However, 20% (6/30) of the participants did not complete the IBSC and stated that reasons for this were often stress (eg, due to obligations at work or in studies) or trouble prioritizing sessions. It is possible that the intervention could need further personalization, for example, in relation to the contact frequency, to be able to accommodate differential needs—a fact also indicated by the qualitative study [31]. However, it cannot be ruled out that ADHD alone or in combination with autism could be the reason for the majority of the dropouts of the intervention group. Several previous intervention studies on individuals with ADHD have reported high dropout rates [68].

### Strengths and Limitations

This study has limitations that need to be considered. First, groups were not randomized due to expected recruitment problems (ie, individuals with ASD have trouble tolerating uncertainty about the arm they will be allocated to, which was a problem in the undertaking of the validation study). It was also deemed unethical to not provide the control group with adequate support during the intervention period. However, this was a controlled study, and the groups were matched as far as possible by age, sex, and diagnosis. Problems with recruitment at the end of the process meant that some exceptions were made but the analysis of demographics and sample characteristics at baseline showed that there were no differences between groups on these parameters. A retrospective GAF assessment revealed that the intervention group had a significantly lower mean GAF score than the comparison group. In line with this, they were less likely to be employed. To assess the feasibility of the intervention, we tried to recruit a naturalistic sample. This meant that recruitment and intervention took place in routine care settings. Inclusion criteria were deliberately generous including individuals with both ASD and ADHD and only excluding those with serious psychiatric comorbidity (if in need of other treatment). It cannot be entirely ruled out that the intervention group was recruited based on perceived need for support to a larger extent than the comparison group and could be a possible explanation for the difference in GAF scores. The difference was statistically adjusted for the analysis.

As the recruited sample was heterogeneous with regard to diagnosis and age, it might limit the extent to which results are applicable to subsamples such as individuals with exclusively ADHD, ASD, or a certain age range. We suspect that some aspects of the format, such as it being text based and delivered in one’s own home, might have appealed more to individuals with ASD. However, the heterogeneity of the sample is also considered a strength of the study, as it is largely representative of a clinical reality. In recent years, this particular topic has been raised within the context of evidence-based medicine [69]. For example, it has been questioned to what extent results from randomized controlled efficacy studies with narrow inclusion criteria, and often high-functioning participants treated within university settings, are generalizable to clinical contexts [69,70]. We seldom see clear-cut case presentations in clinical practice due to the vast overlap among NDDs and their high frequency of comorbidity [9]. Furthermore, it is important to address the needs of individuals with complex case presentations. They may be in particular need of attendance, but there is a deficiency in research for this group [11,71]. It has also been proposed that adding qualitative measures rather than exclusively relying on quantitative self-report questionnaires could increase the clinical relevance of conducted research and offer an important patient perspective [69].

The results of this study were compiled after some time had elapsed. The reason was partly related to practical circumstances and partly due to the fact that a qualitative study pertaining to the same project had been undertaken, analyzed, and published before the quantitative results [31]. With regard to advances in internet-delivered treatment during this intervening period, there has been a surge of studies in this area of research, including some papers covering synchronous text-based interventions [27,72]. However, these have mainly pertained to interventions such as guided or unguided internet-delivered cognitive behavioral therapy for mental health disorders and to a lesser amount interventions specifically targeting individuals with NDDs. Relevant internet-delivered intervention studies pertaining to this specific group are mentioned in the introduction section. None of the interventions were chat based.

### Conclusions

In this IBSC study, an increase in self-esteem and a decrease in anxiety levels were observed 6 months after the intervention. IBSC seems to have significance and is a feasible option for young people with NDDs. There is a need to develop support options that address the obstacles experienced by individuals with NDDs in receiving health care. Future studies should determine in detail for whom and to what degree this kind of support will be regarded as helpful.

## Appendix

Multimedia Appendix 1 Attrition bias analysis.

Multimedia Appendix 2 Primary and secondary outcome measures at baseline, 8 weeks, and 6 months for both study arms (internet-based support and coaching and treatment as usual).

## Abbreviations

ADHD: attention-deficit/hyperactivity disorder
ANCOVA: analysis of covariance
ASD: autism spectrum disorder
CBT: cognitive behavioral therapy
CONSORT: Consolidated Standards of Reporting Trials
CM: clinical management
DSM-IV: Diagnostic and Statistical Manual of Mental Disorders, 4th Edition
GAF: Global Assessment of Functioning
HADS: Hospital Anxiety and Depression Scale
IBSC: internet-based support and coaching
MADRS-S: Montgomery-Åsberg Depression Rating Scale-Self-reported
MANSA: Manchester Short Assessment for Quality of Life
NDD: neurodevelopmental disorder
QoL: quality of Life
RSES: Rosenberg Self-Esteem Scale
SCID I: Structured Clinical Interview for DSM Axis I Disorder
SCID II: Structured Clinical Interview for DSM-IV Axis II Disorder
SOC: Sense of Coherence
TAU: treatment as usual
