# Supplementary material for: Internet-Based Support and Coaching With Complementary Clinic Visits for Young People With Attention-Deficit/Hyperactivity Disorder and Autism: Controlled Feasibility Study
Source: J Med Internet Res. 2020 Dec 31;22(12):e19658. doi: 10.2196/19658 (PMC7808894; doi:10.2196/19658)
Supplement: Multimedia Appendix 1 [file jmir_v22i12e19658_app1.docx]

**Multimedia Appendix 1.** Attrition Bias Analysis of the Intervention group

| **Variable** | **Intervention** n=24 | **Drop-outs** n=6 | ***P*-value** |
| --- | --- | --- | --- |
|  |  |  |  |
| **Age at inclusion**  Mean (SD)  Median (range) | 21.0 (5.1) 20.0 (15.0; 32.0) | 22.2 (4.2) 21.5 (17.0; 29.0) | .62 |
| **Gender, n (%)** |  |  |  |
| Male | 13 (54.2%) | 3 (50.0%) |  |
| Female | 11 (45.8%) | 3 (50.0%) | 1.00 |
|  |  |  |  |
| **Diagnosis, n (%)** |  |  |  |
| ASD | 9 (37.5%) | 2 (33.3%) |  |
| ADHD | 3 (12.5%) | 2 (33.3%) |  |
| ASD + ADHD | 12 (50.0%) | 2 (33.3%) | .46 |
|  |  |  |  |
| **GAF-score (10-point interval)** |  |  |  |
| 21-30 | 0 (0.0%) | 1 (16.7%) |  |
| 31-40 | 9 (37.5%) | 0 (0.0%) |  |
| 41-50 | 9 (37.5%) | 3 (50.0%) |  |
| 51-60 | 5 (20.8%) | 2 (33.3%) |  |
| 61-70 | 1 (4.2%) | 0 (0.0%) | .78 |
|  |  |  |  |
| **Study Centre (1 and 2), n (%)** |  |  |  |
| 1 | 11 (45.8%) | 2 (33.3%) |  |
| 2 | 13 (54.2%) | 4 (66.7%) | .94 |
|  |  |  |  |
| **Geographical area, n (%)** |  |  |  |
| Urban | 13 (54.2%) | 2 (33.3%) |  |
| Rural | 11 (45.8%) | 4 (66.7%) | .65 |
|  |  |  |  |
| **Civil state, n (%)** |  |  |  |
| Married/living with partner | 2 (8.3%) | 1 (16.7%) |  |
| In a relationship (not living together) | 2 (8.3%) | 1 (16.7%) |  |
| Single | 20 (83.3%) | 4 (66.7%) | .66 |
|  |  |  |  |
| **Living situation, n (%)** |  |  |  |
| One person household | 8 (33.3%) | 2 (33.3%) |  |
| Living with partner and/or children | 2 (8.3%) | 1 (16.7%) |  |
| Living with parents and/or siblings | 14 (58.3%) | 2 (33.3%) |  |
| Living with friends and/or acquaintances | 0 (0.0%) | 1 (16.7%) | .18 |
|  |  |  |  |
| **Level of education (completed or ongoing)** |  |  |  |
| Has not completed compulsory school ^a^ | 2 (8.3%) | 2 (33.3%) |  |
| Compulsory School | 7 (29.2%) | 1 (16.7%) |  |
| Upper Secondary School | 11 (45.8%) | 2 (33.3%) |  |
| Vocational education (after compulsory school) | 0 (0.0%) | 1 (16.7%) |  |
| University | 4 (16.7%) | 0 (0.0%) | .31 |
| **Occupation** |  |  |  |
| employed | 0 (0.0%) | 1 (16.7%) |  |
| unemployed | 6 (25.0%) | 3 (50.0%) |  |
| Student ^b^ | 13 (54.2%) | 2 (33.3%) |  |
| work experience placement | 2 (8.3%) | 0 (0.0%) |  |
| sick leave | 3 (12.5%) | 0 (0.0%) | .15 |
|  |  |  |  |
| **Support from social services** ^c^ |  |  |  |
| Yes | 7 (29.2%) | 3 (50.0%) |  |
| No | 17 (70.8%) | 3 (50.0%) | .61 |
|  |  |  |  |
| **ADHD medication at baseline** ^d^ |  |  |  |
| No | 14 (58.3%) | 6 (100.0%) |  |
| Yes | 10 (41.7%) | 0 (0.0%) | .13 |
|  |  |  |  |
| **MANSA Total score Baseline** | 52.1 (9.6) 51.5 (29.0; 71.0) | 53.2 (6.7) 53.0 (43.0; 63.0) | .80 |
| **Subjective quality of life (MANSA) Baseline** ^f^ |  |  |  |
| 2 | 2 (8.3%) | 0 (0.0%) |  |
| 3 | 5 (20.8%) | 2 (33.3%) |  |
| 4 | 7 (29.2%) | 2 (33.3%) |  |
| 5 | 5 (20.8%) | 1 (16.7%) |  |
| 6 | 4 (16.7%) | 0 (0.0%) |  |
| 7 | 1 (4.2%) | 1 (16.7%) | .95 |
|  |  |  |  |
| **Rosenberg Total score Baseline** | 16.2 (5.5) 16.0 (5.0; 28.0) | 18.0 (3.8) 17.5 (13.0; 24.0) | .47 |
| **HADS Anxiety Baseline** | 8.75 (4.30) 8.00 (2.00; 19.00) | 10.3 (5.0) 10.5 (3.0; 17.0) | .45 |
| **HADS Depression Baseline** | 4.50 (3.99) 3.50 (0.00; 13.00) | 6.83 (3.43) 6.50 (2.00; 11.00) | .21 |
| **MADRS-S Total score Baseline** | 13.5 (7.3) 12.0 (1.0; 28.0) | 15.2 (8.6) 17.5 (2.0; 24.0) | .63 |
| **Sense of coherence (SOC) Total score Baseline** | 120.5 (21.4) 118.0 (83.0; 174.0) | 115.9 (14.3) 117.5 (92.0; 130.5) | .62 |

^a^ No formal education or terminated compulsory school without complete grades
^b^ All levels of education, e.g. compulsory school, secondary school, vocational education and university studies  ^c^ Support can include assistance through the Swedish Act concerning Support and Service for Persons with Certain Functional Impairments (LSS) or from Social Services, for example so called “contact person”, relief service or living support

^d^ in all cases but two this was psychostimulant medication

^f^ number of individuals who scored 2, 3, 4, 5, 6, and 7, respectively, on this MANSA item
