# Supplementary material for: Internet-Based Support and Coaching With Complementary Clinic Visits for Young People With Attention-Deficit/Hyperactivity Disorder and Autism: Controlled Feasibility Study
Source: J Med Internet Res. 2020 Dec 31;22(12):e19658. doi: 10.2196/19658 (PMC7808894; doi:10.2196/19658)
Supplement: Multimedia Appendix 2 [file jmir_v22i12e19658_app2.docx]

**Multimedia Appendix 2.** Primary and secondary outcome measures at baseline, 8 weeks and 6 months for both study arms (IBCS and TAU)

|  | **Intervention (IBSC) (n=24)** | | | **Treatment As Usual (TAU) (n=20)** | | |  | | | |
| --- | --- | --- | --- | --- | --- | --- | --- | --- | --- | --- |
| Variable | Mean (SD) Median (Min; Max) | Adjusted Means ^a^ SEM (95% CI) | *P* -value within group | Mean (SD) Median (Min; Max) | Adjusted Means ^a^ SEM (95% CI) | *P* -value within group | | *P* -value between groups | Adjusted *P* –value ^a^ | Difference between groups Adjusted Means (95% CI); Effect size |
|  |  |  |  |  |  |  | |  |  |  |
| **MANSA** |  |  |  |  |  |  |  | |  |  |
| MANSA Total score Baseline | 52.1 (9.6) 51.5 (29.0; 71.0) | 53.7 1.9 (49.8-57.6) |  | 54.8 (10.0) 56.5 (33.0; 69.0) | 52.8 2.1 (48.5-57.1) |  | .37 | | .77 |  |
| Change MANSA Total score Baseline to 8 weeks | 0.522^b^ (8.13) 2.0 (-20.0; 13.0) | 0.294 1.56 (-2.86-3.45) | .58 | -2.25 (5.62) -2.85 (-12.0; 11.0) | -1.99 1.68 (-5.39-1.41) | .058 | .21 | | .35 | 2.28 (-2.57; 7.13); 0.397 |
| Change MANSA Total score Baseline to 6 months | 0.287 (8.93) 1.50 (-17.0; 17.0) | 0.247 1.70 (-3.19-3.68) | .81 | -1.89 (6.33) -3.00 (-13.0; 12.0) | -1.84 1.88 (-5.63-1.96) | .21 | .37 | | .44 | 2.08 (-3.27; 7.44); 0.281 |
| **Subjective quality of life (MANSA)** |  |  |  |  |  |  |  | |  |  |
| Subjective quality of life  Baseline | 4.29 (1.33) 4.00 (2.00; 7.00) | 4.55 0.24 (4.07-5.04) |  | 4.90 (1.21) 5.00 (2.00; 7.00) | 4.58 0.26 (4.05-5.12) |  | .13 | | .94 |  |
| Change Subjective quality of life Baseline to 8 weeks | 0.174^b^ (1.23) 0.00 (-3.00; 3.00) | 0.163 0.258 (-0.358-0.684) | .53 | -0.150 (1.09) 0.00 (-2.00; 3.00) | -0.137 0.278 (-0.700-0.425) | .48 | .44 | | .45 | 0.300 (-0.501; 1.10); 0.279 |
| Change Subjective quality of life Baseline to 6 months | 0.208 (1.22) 0.00 (-2.00; 2.00) | 0.260 0.278 (-0.301-0.821) | .50 | 0.00 (1.376) 0.00 (-2.00; 3.00) | -0.062 0.307 (-0.682-0.558) | .89 | .64 | | .46 | 0.321 (-0.554; 1.20); 0.160 |
| **Rosenberg** |  |  |  |  |  |  |  | |  |  |
| Rosenberg Total score Baseline | 16.2 (5.5) 16.0 (5.0; 28.0) | 17.3 1.0 (15.2-19.4) |  | 18.5 (5.6) 18.5 (7.0; 28.0) | 17.1 1.1 (14.8-19.4) |  | .18 | | .90 |  |
| Change Rosenberg Total score Baseline to 8 weeks | 1.43^b^ (3.30) 1.00 (-5.00; 9.00) | 1.57 0.81 (-0.06-3.20) | .060 | 0.200 (4.06) 0.00 (-8.00; 8.00) | 0.043 0.872 (-1.72-1.81) | .79 | .29 | | .23 | 1.53 (-0.98; 4.04); 0.332 |
| Change Rosenberg Total score Baseline to 6 months | 1.54 (3.59) 2.00 (-8.00; 7.00) | 1.29 0.81 (-0.36-2.93) | .038 | 0.050 (4.07) 0.00 (-6.00; 10.0) | 0.356 0.900 (-1.46-2.17) | .86 | .21 | | .47 | 0.930 (-1.64; 3.49); 0.388 |
| **HADS ANX** |  |  |  |  |  |  |  | |  |  |
| HADS Anx Baseline | 8.75 (4.30) 8.00 (2.00; 19.00) | 8.02 0.83 (6.34-9.69) |  | 7.05 (4.14) 6.00 (0.00; 17.0) | 7.93 0.91 (6.08-9.78) |  | .19 | | .95 |  |
| Change HADS Anx Baseline to 8 weeks | -0.435^b^ (2.56) -1.00 (-7.00; 5.00) | -0.432 0.616 (-1.68-0.813) | .44 | 1.80 (3.04) 1.00 (-3.00; 11.0) | 1.80 0.67 (0.45-3.14) | .010 | .012 | | .024 | -2.23 (-4.15; -0.31); 0.796 |
| Change HADS Anx Baseline to 6 months | -1.87^b^ (2.69) -2.00 (-8.00; 3.00) | -1.52 0.66 (-2.85--0.20) | .003 | 1.95 (3.41) 2.00 (-3.00; 10.0) | 1.55 0.71 (0.12-2.99) | .023 | .001 | | .004 | -3.08 (-5.14; -1.02); 1.24 |
| **HADS DEPR** |  |  |  |  |  |  |  | |  |  |
| HADS Depr Baseline | 4.50 (3.99) 3.50 (0.00; 13.00) | 3.93 0.67 (2.57-5.29) |  | 3.80 (2.53) 4.00 (0.00; 10.0) | 4.48 0.74 (2.98-5.99) |  | .51 | | .60 |  |
| Change HADS Depr Baseline to 8 weeks | -0.435^b^ (2.35) -1.00 (-7.00; 5.00) | -0.502 0.625 (-1.77-0.760) | .41 | 1.50 (3.30) 0.00 (-2.00; 11.0) | 1.58 0.67 (0.22-2.94) | .063 | .030 | | .036 | -2.08 (-4.02; -0.14); 0.675 |
| Change HADS Depr Baseline to 6 months | -0.174^b^ (2.85) -1.00 (-6.00; 5.00) | -0.081 0.643 (-1.38-1.22) | .80 | 1.25 (2.94) 1.00 (-3.00; 10.0) | 1.14 0.70 (-0.26-2.55) | .091 | .12 | | .23 | -1.22 (-3.24; 0.79); 0.491 |
| **MADRS-S** |  |  |  |  |  |  |  | |  |  |
| MADRS-S Total score Baseline | 13.5 (7.3) 12.0 (1.0; 28.0) | 12.0 1.4 (9.2-14.8) |  | 10.5 (7.2) 9.0 (2.0; 29.0) | 12.2 1.5 (9.1-15.3) |  | .19 | | .94 |  |
| Change MADRS-S Total score Baseline to 8 weeks | 0.043^b^ (6.09) 0.00 (-14.0; 15.0) | -0.103 1.26 (-2.66-2.45) | .96 | 1.59 (5.28) 1.00 (-11.0; 12.0) | 1.76 1.36 (-1.00-4.51) | .23 | .39 | | .34 | -1.86 (-5.79; 2.07); 0.272 |
| Change MADRS-S Total score Baseline to 6 months | -0.833 (6.47) 0.500 (-13.0; 15.0) | -0.243 1.45 (-3.18-2.70) | .62 | 1.75 (7.33) 2.00 (-12.0; 21.0) | 1.04 1.61 (-2.20-4.29) | .33 | .23 | | .57 | -1.28 (-5.86; 3.30); 0.374 |
| **Sense of coherence (SOC)** |  |  |  |  |  |  |  | |  |  |
| SOC  Total Baseline | 120.5 (21.4) 118.0 (83.0; 174.0) | 125.0 4.1 (116.7-133.2) |  | 126.7 (22.4) 132.0 (77.0; 162.0) | 121.2 4.5 (112.1-130.4) |  | .36 | | .56 |  |
| Change SOC Total Baseline to 8 weeks | 1.30^b^ (12.9) 3.00 (-24.0; 34.0) | 2.02 3.13 (-4.32-8.35) | .58 | -1.95 (15.74) -5.00 (-24.0; 38.0) | -2.77 3.38 (-9.60-4.07) | .14 | .47 | | .33 | 4.78 (-4.96; 14.5); 0.226 |
| Change SOC Total Baseline to 6 months | 6.33 (16.7) 5.00 (-22.0; 41.0) | 5.81 3.18 (-0.62-12.2) | .10 | -4.02 (12.0) -0.71 (-27.9; 13.0) | -3.39 3.52 (-10.5-3.71) | .19 | .026 | | .071 | 9.19 (-0.83; 19.2); 0.712 |
| ^a^Adjusting for GAF intervall using Analysis of Covariance (ANCOVA)  ^b^ Based on 23 individuals  *SD* standard deviation, *SEM* Standard error of the mean, *CI* confidence interval, *MANSA* Manchester Short Assessment of Quality of Life, *Rosenberg* Rosenberg Self-Esteen Scale, *HADS* Hospital Anxiety and Depression Scale, *MADRS-S* Montgomery-Åsberg Depression Rating Scale - Self-reported, *SOC* Sense of Coherence, *Baseline* time of inclusion | | | | | | | | | | |
